# Peripheral arterial occlusive disease: Global gene expression analyses suggest a major role for immune and inflammatory responses

**DOI:** 10.1186/1471-2164-9-369

**Published:** 2008-08-01

**Authors:** Shijun Fu, Haiguang Zhao, Jiantao Shi, Arhat Abzhanov, Keith Crawford, Lucila Ohno-Machado, Jianqin Zhou, Yanzhi Du, Winston Patrick Kuo, Ji Zhang, Mier Jiang, Jason Gang Jin

**Affiliations:** 1 Institute of Health Sciences, Shanghai Institutes for Biological Sciences, Chinese Academy of Sciences and Shanghai Jiao Tong University School of Medicine, Shanghai, PR China; 2 Shanghai Ninth People's Hospital, Shanghai Jiao Tong University School of Medicine, Shanghai, PR China; 3 Laboratory for Innovative Translational Technologies, Harvard School of Dental Medicine, Boston, MA, USA; 4 ShanghaiBio Corporation, 675 US Highway One, North Brunswick, NJ, USA; 5 Shanghai Biochip Co., Ltd and National Engineering Center for Biochip at Shanghai, Shanghai, PR China; 6 Department of Organismic and Evolutionary Biology, Harvard University, Cambridge, MA, USA; 7 Department of Developmental Biology, Harvard School of Dental Medicine, Boston, MA, USA; 8 Decision Systems Group, Brigham and Women's Hospital, Harvard Medical School, Boston, MA, USA; 9 Department of Orthopedics, Brigham and Women's Hospital, Harvard Medical School, Boston, MA, USA; 10 School of Pharmacy, Soochow University, Suzhou, JiangSu, PR China; 11 Graduate School of the Chinese Academy of Sciences, Shanghai, PR China

## Abstract

**Background:**

Peripheral arterial disease (PAD), a major manifestation of atherosclerosis, is associated with significant cardiovascular morbidity, limb loss and death. However, mechanisms underlying the genesis and progression of the disease are far from clear. Genome-wide gene expression profiling of clinical samples may represent an effective approach to gain relevant information.

**Results:**

After histological classification, a total of 30 femoral artery samples, including 11 intermediate lesions, 14 advanced lesions and 5 normal femoral arteries, were profiled using Affymetrix microarray platform. Following real-time RT-PCR validation, different algorithms of gene selection and clustering were applied to identify differentially expressed genes. Under a stringent cutoff, i.e., a false discovery rate (FDR) <0.5%, we found 366 genes were differentially regulated in intermediate lesions and 447 in advanced lesions. Of these, 116 genes were overlapped between intermediate and advanced lesions, including 68 up-regulated genes and 48 down-regulated ones. In these differentially regulated genes, immune/inflammatory genes were significantly up-regulated in different stages of PAD, (85/230 in intermediate lesions, 37/172 in advanced lesions). Through literature mining and pathway analysis using different databases such as Gene Ontology (GO), and the Kyoto Encyclopedia of Gene and Genomics (KEGG), genes involved in immune/inflammatory responses were significantly enriched in up-regulated genes at different stages of PAD(p < 0.05), revealing a significant correlation between immune/inflammatory responses and disease progression. Moreover, immune-related pathways such as Toll-like receptor signaling and natural killer cell mediated cytotoxicity were particularly enriched in intermediate and advanced lesions (P < 0.05), highlighting their pathogenic significance during disease progression.

**Conclusion:**

Lines of evidence revealed in this study not only support previous hypotheses, primarily based on studies of animal models and other types of arterial disease, that inflammatory responses may influence the development of PAD, but also permit the recognition of a wide spectrum of immune/inflammatory genes that can serve as signatures for disease progression in PAD. Further studies of these signature molecules may eventually allow us to develop more sophisticated protocols for pharmaceutical interventions.

## Background

Peripheral arterial occlusive disease (PAD) is a major manifestation of atherosclerosis and is commonly found in elderly patients. Epidemiological studies have shown that PAD affects 8 to 10 million adults in the United States [1]. Most patients with PAD are asymptomatic. The disease is primarily diagnosed by an ankle brachial index (ABI) < 0.9. The most common symptom of mild-to-moderate PAD is intermittent claudication, which is present in about one third of symptomatic patients [1]. In addition to leg symptoms, patients with PAD are at an increased risk for developing new coronary events and eventually death from cardiovascular disease. Although conventional procedures such as stents, arterectomies, angioplasty, and bypass surgery have been successful in improving clinical symptoms of PAD to a large extent [2], ultimately elimination of the disease may require sophisticated protocols of pharmaceutical interventions, which may depend on better understanding of molecular mechanisms involved in the disease.

Previous studies have implicated the involvement of the immune system in atherosclerosis formation and progression. Animal models have been used to test the contributions of components of the immune system [3,4]. Cellular involvement of macrophages was found to be important in the formation and progression of atherosclerosis in animal models [4]. In addition, various immune-related genes have been examined in an atherosclerosis animal model, and genes such as *CXCR6, CXCL10, CXCR3 *and *CXCL16/scavenger receptor *have been shown to be involved in the progression of atherosclerosis in animal models [5-8]. In humans, many immune cells such as macrophages, lymphocytes, mast cells, and T cells are found in atherosclerosis [9]. These findings suggest that the immune system plays important roles in atherogenesis. However, data available to date are primarily derived from studies of atherosclerosis in the coronary or/and the carotid arteries, whereas data derived from clinical samples of PAD appear to be particularly limited.

In the past decade, microarray analysis using high-throughput screening technology has emerged as an important tool to study gene expression patterns and to study molecular events in complex diseases [10-12]. In this study, Affymetrix GeneChips were used to perform gene expression profiling of femoral atherosclerotic lesions to fully characterize the peripheral arterial wall gene expression patterns associated with atherosclerosis. By statistical analysis, hundreds of known and novel genes were identified that differentially express in PAD. Genes involved in immune/inflammatory responses appeared to be significantly enriched in the set of genes up-regulated in different stages of PAD. To further examine the expression patterns of individual genes in the context of particular biological or molecular pathways, gene functional enrichment was performed using Gene Ontology and KEGG database. The results revealed that immune system-related categories and pathways were significantly overrepresented in the progression of the disease, suggesting that up-regulation of immune/inflammatory genes may be critical components of the disease progression expression signature associated with atherosclerosis. These findings may provide new insights and foster a better understanding of the mechanism of PAD.

## Results

### Patient classification and outcome

**Figure 1:**
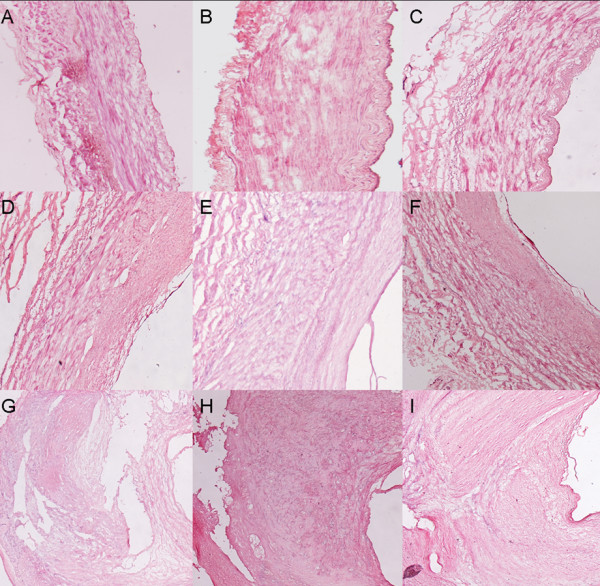
**Histological characteristics of various severities of femoral atherosclerotic lesions in PAD patients**. HE stain analysis of histological characteristics of collected femoral non-atherosclerotic arteries and atherosclerotic arteries. 8 um cryostat sections were stained with hematoxylin and eosin, dehydrated in graded alcohol, and cover-slipped with permanent mounting solution after xylene clearing. Three representative samples are listed: normal artery (A, B, C), intermediate lesions (D, E, F), and advanced lesions (G, H, I).

Histological characterization of 30 collected peripheral artery samples was conducted based on the criteria of the American Heart Association. Of these samples, 15 were classified as grade III (intermediate lesions), one as grade IV and fourteen as grade V (advanced lesions). Among them, 11 intermediate lesions samples (grade III) and 14 advanced lesions samples (grade V) had RNA of sufficient quality and quantity for hybridization. Representative images of the different stages are shown in Figure 1. Further details of these 25 samples are listed in Table 1. As shown, there was no significant difference between the intermediate lesions and the advanced lesions group except for indications of hypertension. In the intermediate lesions group, 4 patients (36.4%) presented with hypertension, while 9 hypertensive patients (64.3%) were found in the advanced lesions group.

**Table 1:** Patient characteristics for the 25 samples in the microarray analysis

	**Intermediate lesions group (n = 11)**	**Advanced lesions group (n = 14)**
Age*	76.1 ± 4.68	76.6 ± 3.79
Male, n (%)	8(72.7)	12(85.7)
Hypertension^†^, n (%)		
Yes	4(36.4)	9(64.3)
no	7(63.6)	5(35.7)
Hypercholesterolemia, n (%)		
Yes	9(81.8)	11(78.6)
no	2(18.2)	3(21.4)
Diabetes, n (%)		
Yes	7(63.6)	10(71.4)
no	4(36.4)	4(28.6)

*as mean ± SD.

† P < 0.05 (Student t test)

### Differentially regulated genes in intermediate lesions

**Figure 2:**
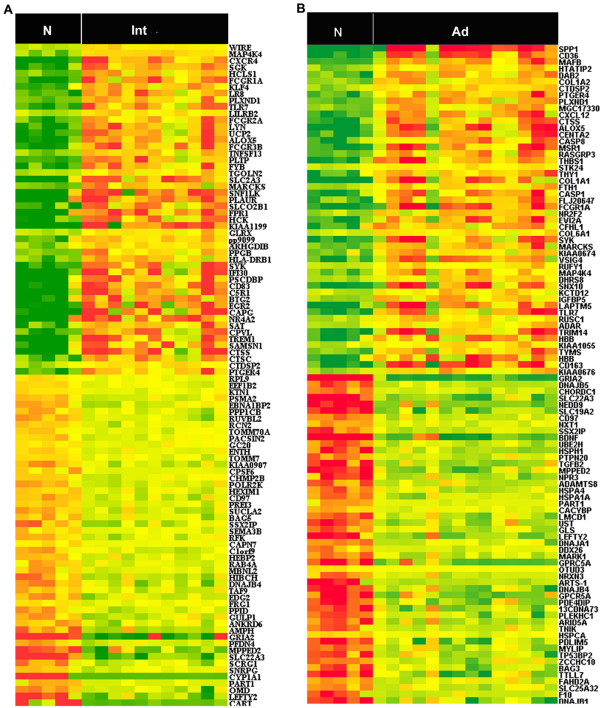
**Heatmap of the 100 most differentially expressed genes in intermediate lesions and advanced lesions, respectively**. SAM analysis reveals genes with differential expression in PAD. This analysis compared plaques from within arteries of either intermediate (n = 11) or advanced lesions (n = 14) to normal control group, respectively. Heatmap representation of the 100 most differentially expressed in intermediate lesions (A) and advanced lesions (B). Samples are displayed in columns and genes in rows. Gene expression is represented as a color, normalized across each row, with brighter red for higher values and brighter green for lower values. Gene symbols are listed to the right. N (Normal control group), Int (intermediate lesions group), Ad(advanced lesions group). The list of differentially expressed genes in intermediate lesions and advanced lesions is provided [see Additional file 1 and Additional file 4].

Identifying differential expression genes was achieved for different stages by using Significance Analysis of Microarrays (SAM) with a false discovery rate (FDR) of 0.5%. Comparative analysis revealed that 366 genes were differentially expressed in intermediate lesions when compared to normal femoral arteries, of which 230 genes were up-regulated and 136 were down-regulated [see Additional file 1]. The 100 most differentially expressed genes between intermediate lesions and normal femoral arteries are shown in Figure 2A. Notably, in the up-regulated genes, up to 85 genes have been reported to be involved in immune response, such as *HLA-DQB1, HLA-DRB1, CCR1, CXCR4, C1QB *and *TLR7 *[see Additional file 2]. In addition, a large number of genes known to encode proteins crucial for proteolysis (*CTSB, CTSC, CTSD and CTSS*) and cell proliferation (*BTG1, BTG2, CDKN1A, and MCM5*) appeared to be significantly changed. Since BTG1 and BTG2 are known to be involved in anti-proliferation activities, it can be of interest to further investigate their potential roles in PAD in detail. MCM5 is heavily involved in chromosomal stability. Among the down-regulated genes, those involved in calcium signaling (*CAMK2G)*, transport (*SLC22A3, CYP1A1 *and *ATP5H*), metabolism (*GCSH *and *PLA2G4A*), and protein amino acid dephosphorylation (*PTPN20*) were found to be significantly down-regulated. The Gene Ontology functional categories in which intermediate lesions are overrepresented are illustrated in Table 2 and additional data [see Additional file 3]. As shown in the table, the most significant biological process categories in the up-regulated genes are immune response, humoral immune response, inflammatory response, and T cell proliferation (Z-score>5). For down-regulated genes, the significant ones mainly represent metabolism and catabolism-related categories.

**Table 2:** Biological process categories overrepresented in intermediate lesions

**GO Name**	**Z-score**	***P*-value**
**For up-regulated genes **		

immune response	14.491	0.000
antigen processing, endogenous antigen via MHC class I	3.821	0.014
antigen processing, exogenous antigen via MHC class II	5.968	0.001
cellular defense response	5.669	0.000
cell-mediated immune response	3.093	0.038
T-helper 1 type immune response	3.214	0.035
humoral immune response	9.451	0.000
complement activation	3.608	0.016
inflammatory response	8.515	0.000
neutrophil chemotaxis	6.629	0.004
leukocyte adhesion	4.767	0.013
immune cell activation	3.268	0.011
immune cell migration	6.25	0.000
cell surface receptor linked signal transduction	2.082	0.042
integrin-mediated signaling pathway	2.633	0.035
intracellular signaling cascade	4.367	0.000
MAPKKK cascade	2.413	0.037
myeloid cell differentiation	2.876	0.042
lipid transport	3.255	0.011
Phagocytosis	4.757	0.005
Apoptosis	2.091	0.049
cell proliferation	4.257	0.000
T cell proliferation	5.489	0.003
oxygen transport	3.255	0.016
icosanoid metabolism	3.524	0.010
lipoprotein metabolism	3.291	0.017

**For down-regulated genes**		

cellular catabolism	2.708	0.014
amine catabolism	3.599	0.011
glycine catabolism	8.457	0.003
glycine metabolism	5.817	0.008
ubiquitin cycle	2.092	0.041
protein folding	3.088	0.008
Endocytosis	2.521	0.032

GO analysis was applied to differentially expressed genes in intermediate lesions compared with normal controls. Similar significant categories were not included to reduce redundancy. The first half of the table indicates categories highly significant for up-regulated genes; the second half of the table shows categories highly significant for down-regulated genes. Calculated *p*-values and Z-scores for each category are shown. The corresponding cell component categories and molecular function categories are available in additional data [see Additional file 3]

### Differentially regulated genes in advanced lesions

When advanced lesions were compared to normal femoral arteries, 447 genes were identified, of which 172 genes were up-regulated and 275 were down-regulated [see Additional file 4]. The list of the 100 most differentially expressed genes is shown in Figure 2B. Interestingly, up to 37 genes involved in the immune system response, such as *CCR1, CX3CR1, TLR1 and TLR7*, were found to be up-regulated in advanced lesion [see Additional file 5], which might suggest that these immune/inflammatory related genes could serve as expression signatures characterizing different stages of PAD. In addition, genes constituting a major portion of the vascular extracellular matrix were significantly up-regulated in advanced lesions, including *COL1A1, COL3A1, COL1A2, COL5A1, COL6A1, COL6A3 and LAMB1*, suggesting that these genes could be involved in the femoral artery occlusion in PAD. GO analysis further confirmed the above findings, by highlighting categories of immune response, humoral immune response, inflammatory response and I-kappaB kinase/NF-kappaB cascades (Z-score>5) (Table 3 and Additional file 6). For down-regulated genes, those involved in ion transport (*GRIA2 and SLC22A3*) and protein folding (*DNAJB5*) appeared to be the most significantly down-regulated in advanced lesions. GO analysis showed that the most significant categories for down-regulated genes were response to protein stimulus, RNA metabolism, and protein folding (Table 3 and Additional file 6).

**Table 3:** Biological process categories overrepresented in advanced lesions

**GO Name**	**Z-score**	***P*-value**
**For up-regulated genes **		

immune response	6.305	0.000
cellular defense response	2.94	0.026
humoral immune response	5.392	0.000
inflammatory response	6.179	0.000
immune cell migration	4.778	0.014
intracellular signaling cascade	4.297	0.001
small GTPase mediated signal transduction	2.372	0.024
I-kappaB kinase/NF-kappaB cascade	6.408	0.000
T cell proliferation	4.186	0.016
induction of apoptosis	3.709	0.007
oxygen transport	4.778	0.003
amine transport	3.051	0.024
anion transport	3.373	0.006
phosphate transport	6.139	0.000
organic acid transport	2.634	0.039
nucleoside monophosphate metabolism	5.853	0.001
cell adhesion	3.958	0.000

**For down-regulated genes**		

response to protein stimulus	6.79	0.000
response to unfolded protein	6.79	0.000
integrin-mediated signaling pathway	4.36	0.003
Apoptosis	2.838	0.003
muscle cell differentiation	3.771	0.032
vasculature development	3.795	0.005
neuron differentiation	3.993	0.003
vitamin transport	3.771	0.023
RNA metabolism	3.476	0.000
RNA splicing	3.497	0.005
glutamine metabolism	4.901	0.004
macromolecule metabolism	2.214	0.023
protein folding	5.643	0.000
cell-matrix adhesion	2.605	0.036
peptide hormone secretion	5.401	0.004

GO analysis was applied to differentially expressed genes in advanced lesions compared with normal controls. Some other selected categories are shown. The first half of the table indicates categories highly significant for up-regulated genes; the second half of the table shows categories highly significant for down-regulated genes. Calculated *p*-values and Z-scores for each category are shown. The corresponding cell component categories and molecular function categories are available in additional data [see Additional file 6]

**Figure 3:**
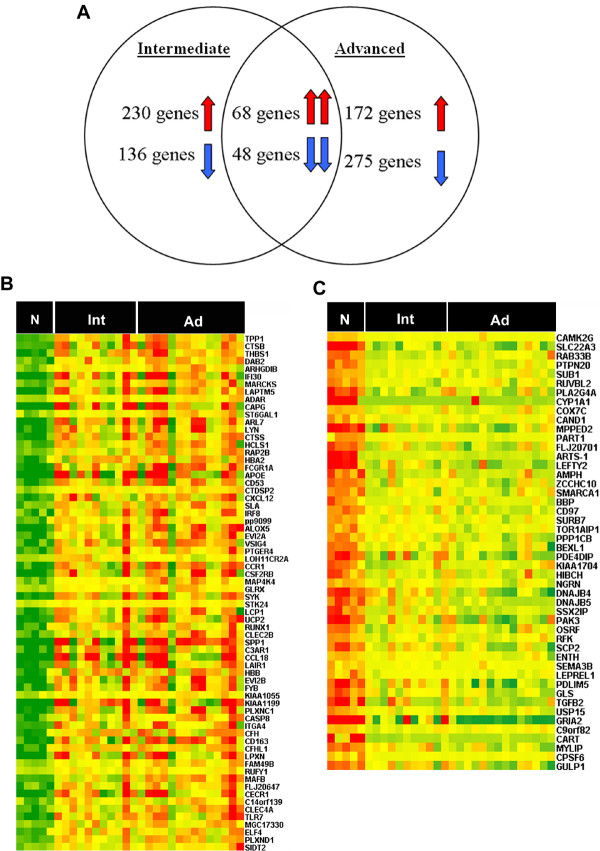
**Over-represented genes in both intermediate lesions and advanced lesions**. A. The genes whose expressions were significantly changed in intermediate lesions and advanced lesions, respectively, are shown in a Venn diagram. B, C. Heatmap representation of commonly up-regulated genes (B) and commonly down-regulated genes (C) in overlapping genes, respectively. Samples are displayed in columns and genes in rows. Gene expression is represented as a color, normalized across each row, with brighter red for higher values and brighter green for lower values. Gene symbols are listed to the right. N (Normal control group), Int (intermediate lesions group), Ad (advanced lesions group).

In parallel, further data analysis revealed that many genes were over-represented in both intermediate and advanced lesions vs. normal controls. Of these genes, 68 were found to be commonly up-regulated and 48 were found to be commonly down-regulated (Figure 3). The list of commonly up-regulated genes is available [see Additional file 7]. Some of these overlapping genes, such as *CTSB, CCR1, ALOX5, and SPP1*, have been previously reported to play important roles in atherogenesis [13-16]. Accordingly, these commonly regulated genes can therefore be important for the progression of PAD. In contrast, a much larger number of genes appear to be characteristically expressed in either intermediate lesions or advanced lesions, which may therefore serve as stage-specific signatures of PAD.

### Differential gene expression in disease progression

**Figure 4:**
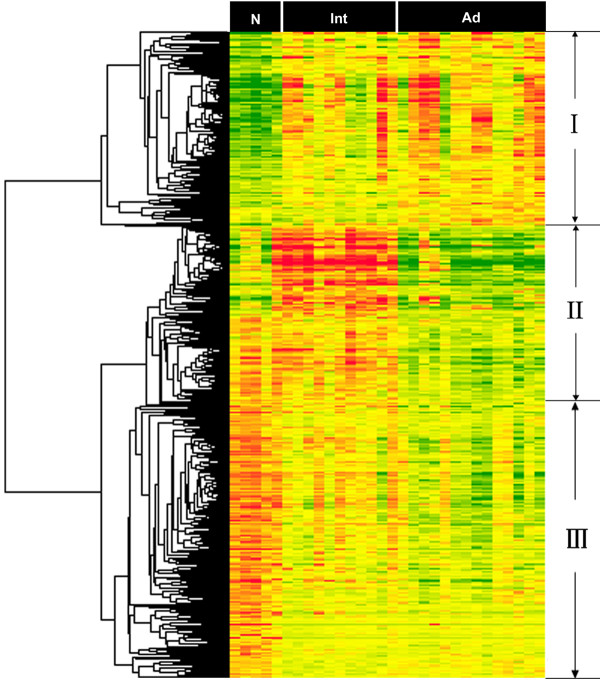
**Hierarchical clustering analysis of the differentially expressed genes in disease progression**. The differentially expressed genes analyzed by hierarchical clustering method in disease progression. The genes were classified into three major clusters by visual inspection. Clustering method: Average linking; Similarity measure: Euclidean distance. Samples are displayed in columns and genes in rows. Gene expression is represented as a color, normalized across each row, with brighter red for higher values and brighter green for lower values. N (Normal control group), Int (intermediate lesions group), Ad (advanced lesions group). The list of differentially expressed genes in disease progression is provided [see Additional file 8].

Intermediate lesions and advanced lesions represent different stages in disease progression of PAD. Identification of genes that exhibit characteristic expression patterns in different stages may provide information relevant to the progression of PAD. For this reason, expression profiles of normal arteries, intermediate lesions and advanced lesions were analyzed by the SAM multiclass method. Out of this analysis, 614 genes appeared to be differentially expressed in the progression of PAD with a FDR<0.5% [see Additional file 8]. Hierarchical clustering analysis suggested that the expression patterns of the genes could be assigned to three major groups (Figure 4). The first group represents those genes commonly expressed in both intermediate and advanced lesions (Cluster II). GO terms indicate that these genes are mainly involved in the immune response, inflammatory response, cellular defense and various signaling pathways (Table 4 and Additional file 9). These results further support the notion that the immune system may play an important role in the progression of PAD. The second group represents specifically down-regulated genes in advanced lesions (Cluster II). GO terms indicate that these genes are primarily involved in cell cycle, apoptosis, multicellular organism development and protein folding (Table 4 and Additional file 9). Genes in the third group are represented by those down-regulated in both intermediate lesions and advanced lesions (Cluster III). GO terms indicate that these genes are mainly involved in neurogenesis, protein modification, RNA splicing, and blood pressure regulation (Table 4 and see Additional file 9). In addition, we have performed data analysis restricted to male subjects. Up to 85% genes identified in male subjects are the same as those identified in the total samples (data not shown), which suggests that the potential gender-biases is minimal. Taken together, genes commonly up-regulated in intermediate and advanced stages are typically represented by those involved in immune and inflammatory responses, implicating enhanced immune response activities during the progression of the disease, whereas down-regulated genes in the both disease stages are primarily represented by those involved in various aspects of cell proliferation and differentiation.

**Table 4:** GO discovered categories for disease progression manner analysis

**GO Name**	**Z-Score**	***P*-value**
**For Cluster I**		

immune response	8.908	0.000
inflammatory response	7.643	0.000
cellular defense response	5.938	0.001
chemotaxis	7.611	0.000
cell surface receptor linked signal transduction	2.937	0.009
cytokine and chemokine mediated signaling pathway	4.378	0.004
JAK-STAT cascade	3.254	0.020
MAPKKK cascade	3.062	0.006
endocytosis	1.956	0.049
angiogenesis	4.452	0.000
cell adhesion	4.580	0.000
ion homeostasis	3.090	0.010

**For Cluster II**		

apoptosis	3.871	0.002
cell cycle	4.108	0.000
cell growth	2.285	0.037
epidermal cell differentiation	3.075	0.044
protein folding	3.677	0.001
macromolecule metabolic process	3.022	0.005
RNA metabolic process	5.378	0.000
transcription	4.593	0.000
peripheral nervous system development	2.950	0.046
multicellular organismal development	3.927	0.000
vitamin transport	4.426	0.008
response to stimulus	4.579	0.000
circadian rhythm	5.090	0.001

**For Cluster III**		

neurogenesis	2.612	0.014
neuron recognition	3.315	0.046
cell motility	2.093	0.042
glycogen metabolic process	2.999	0.025
protein modification process	3.166	0.001
RNA processing	2.183	0.042
RNA splicing	2.591	0.021
phosphate metabolic process	2.346	0.024
blood pressure regulation	4.028	0.008
heart contraction	2.901	0.022

GO analysis was applied to differentially expressed genes in each cluster group. Some selected categories are shown. The Calculated *p*-values and Z-scores for each category are shown. The detailed GO results are provided in the additional data [see Additional file 9].

### Validation of gene transcription by real-time PCR

**Figure 5:**
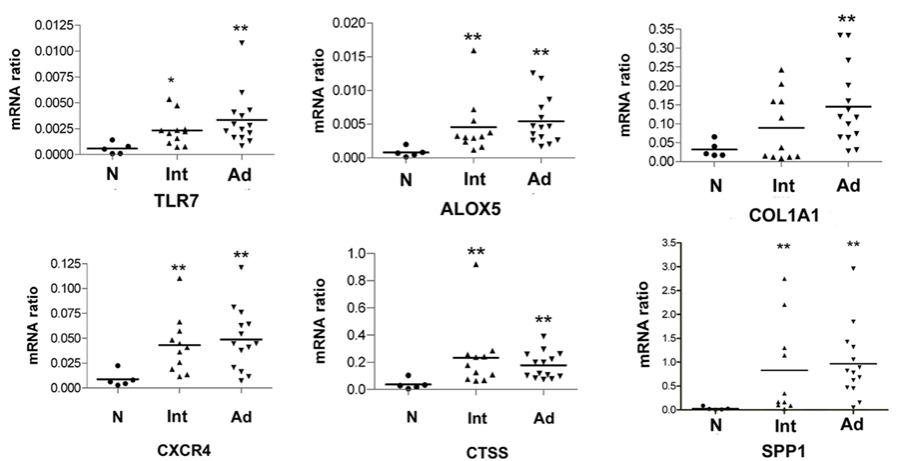
**Real-time PCR and the relative expression level of six genes**. Six genes mRNA in normal femoral artery (N, black round), intermediate lesions (Int, ascending triangle) and advanced lesions (Ad, descending triangle) were determined by real-time PCR and presented as a ratio to GAPDH mRNA. mRNA abundance in intermediate lesions or advanced lesions was differentially expressed (*P < 0.05, and **P < 0.01, respectively) when normal samples were used for comparison.

Real-time PCR is still the gold standard for quantitative analysis of mRNA. In order to validate the microarray results, RT- PCR was carried out on the same set of samples that were analyzed by the microarray approach. The results were highly correlated with those from the array data. (The correlation coefficient for microarray and RT-PCR was 0.835 ± 0.076). Representative RT-PCR results of 6 genes are shown in Figure 5.

### Transcription factors enrichment analysis

**Figure 6:**
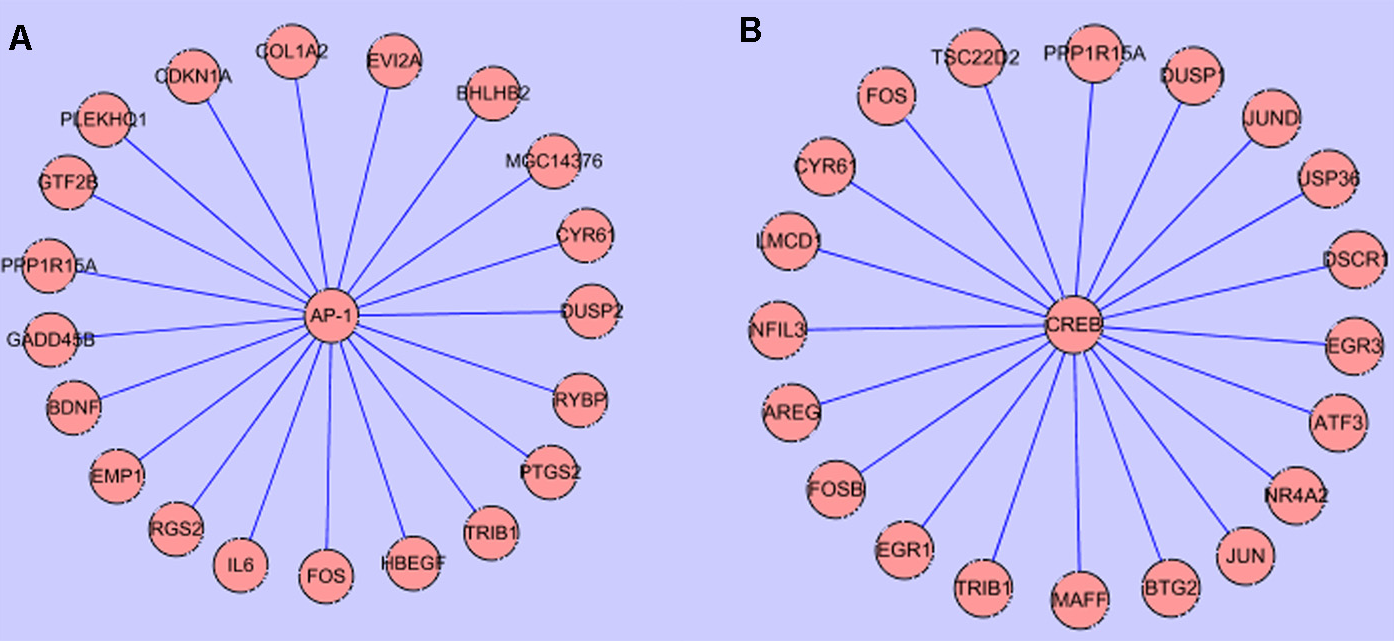
**Enriched transcription factors and their putative target genes in disease progression**. Putative targets of transcription factors were curated based on results by Xiaohui Xie. Fisher Exact test showed that two transcription factors (AP-1 and CREB) were significantly enriched in disease progression (q-value <0.05). AP-1 and CREB were enriched to regulate 72 and 55 genes expression, respectively. A, B. The top 20 putative target genes of AP-1 and CREB were listed, respectively. A list of the enriched transcription factor and their putative targets is provided [see Additional file 10 and Additional file 11].

Transcription factors appear to play important roles in the development or progression of atherosclerosis [17,18]. To address whether specific transcription factors are involved in the regulation of genes associated with the progression of PAD, we conducted a transcription factor binding site enrichment study by analyzing cross-species conserved binding sites in promoter regions of genes differentially regulated during progression of PAD. Through the Fisher Exact test, binding sites of transcription factor AP-1 and CREB appeared to be significantly enriched (*q-value *< 0.05). AP-1 is a transcription factor known to be involved in various cellular processes. In atherosclerosis, it has been reported in gene regulation of microphages, vascular smooth muscle cells and epithelial cells [19,20]. In disease progression, AP-1 was enriched to regulate expression of 72 genes (Figure 6A and Additional file 10). The enrichment of AP1 binding sites in regulated genes associated with PAD progression may therefore suggest an important role played by this transcription factor in the development of PAD. Through literature mining, indeed, some of the potential targets of AP1 appear to be previously reported as target genes of AP-1 [21-24]. CREB is a member of the leucine zipper family of DNA binding proteins. This transcription factor binds as a homodimer to the cAMP-responsive element and induces transcription of genes in response to hormonal stimulation [25]. A total of 55 genes were recognized as potential targets of CREB (Figure 6B and Additional file 11). Although further studies are need to elucidate detailed roles played by AP1 and CREB in PAD progression, significantly enriched binding sites and highly correlated with signature genes of PAD progression suggest that these two transcription factors may play critical roles in the development of PAD.

### Pathways identification by overabundant genes

**Figure 7:**
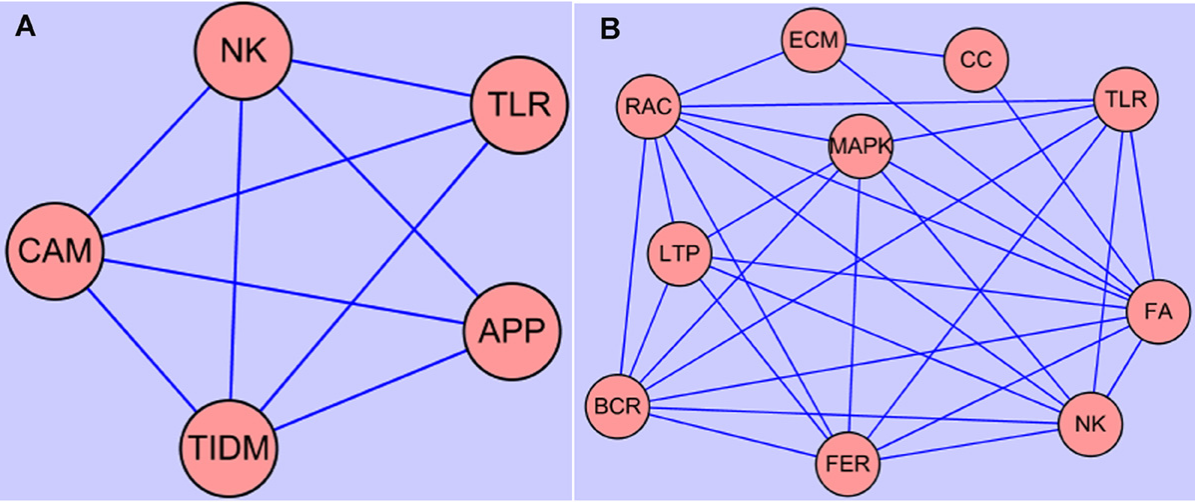
**Interactions of KEGG pathways for differentially expressed genes in different stages of PAD**. Pathways are enriched in intermediate lesions (A) and advanced lesions (B), respectively. Many genes involved in one pathway could also be involved in another pathway. A, B. Networking displayed the interaction of pathways in intermediate lesions and advanced lesions, respectively.

**Figure 8:**
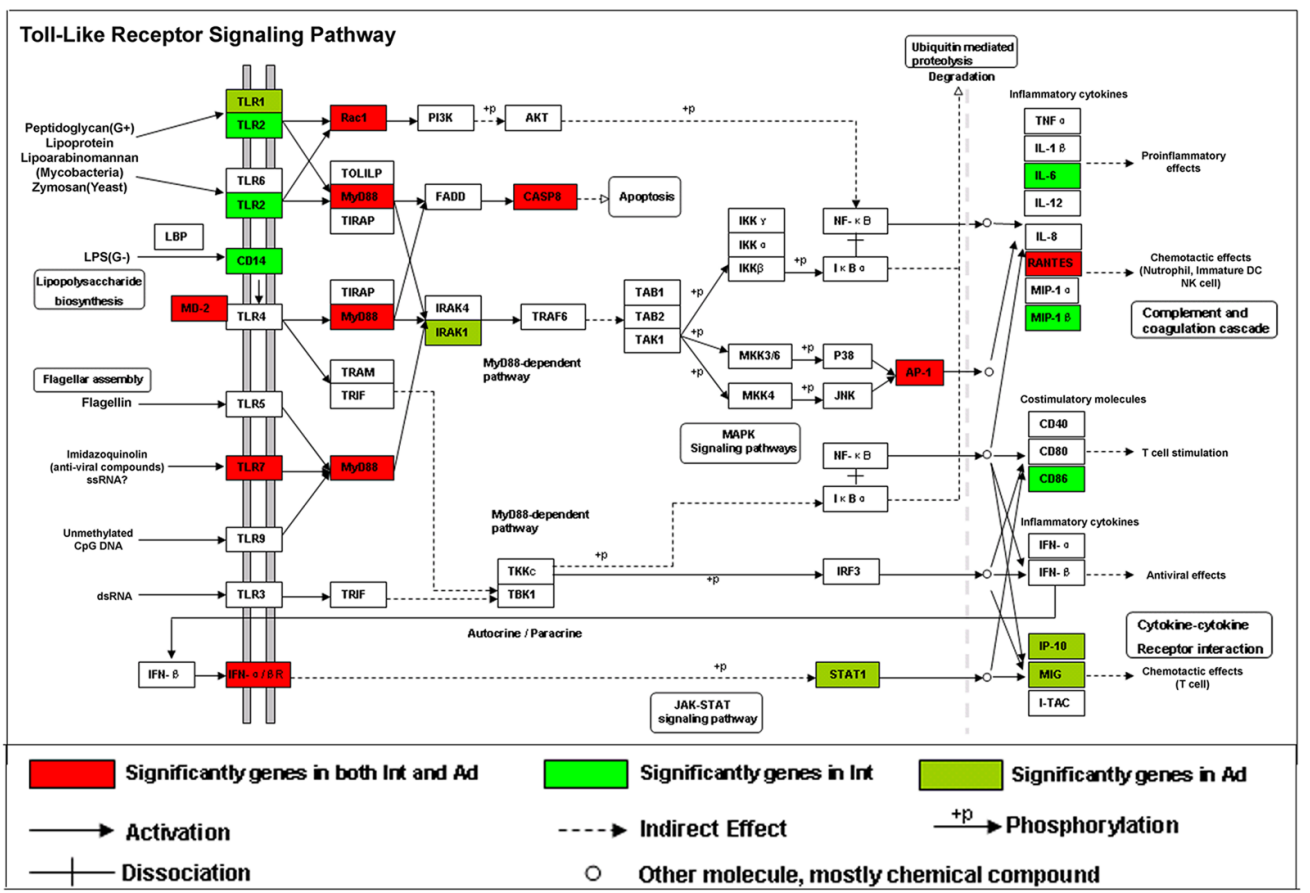
**Over-representation of Toll-like receptor signaling pathway genes**. Analysis of over-representation of differentially expressed genes in pathway from KEGG. The Toll-like receptor signaling pathway is illustrated with significantly regulated genes highlighted.

A pathway analysis database, KEGG, was then applied to genes differentially regulated in intermediate and advanced lesions. Several overrepresented pathways were identified, and the enriched pathways appeared not to be independent of one another, many genes involved in one pathway could be also involved in another pathway. This interaction is illustrated in Figure 7, and pathway abbreviations can be found in Table 5. As demonstrated, many immune-related pathways were significantly over-represented in intermediate and/or advanced lesions including TLR, NK, BCR, FER, APP, CCC and LTEM pathways. These findings, on the one hand, provide evidence supporting previous hypotheses that immune/inflammatory responses play important roles in the development of PAD, and on the other hand, demonstrate that particular components of immune/inflammatory systems can be crucial for the genesis and progression of PAD. For instance, TLR and NK pathways are shown to be particularly overrepresented in both intermediate lesions and advanced lesions, highlighting their functional importance in the disease. The TLR pathway is shown in Figure 8 with the differentially regulated genes indicated.

**Table 5:** KEGG biological pathways for differentially expressed genes in different stages

**KEGG Pathway Name**	**Pathway ID**	**Genes involved **	***P*-Value**
**For intermediate lesions**			

Type I Diabetes Mellitus(TIDM)	Hsa04940	9	0.002
Antigen Processing and Presentation(APP)	Hsa04612	13	0.003
Complement and Coagulation Cascades(CCC)	Hsa04610	12	0.003
Cell Adhesion Molecules(CAM)	Hsa04514	17	0.005
Toll-Like Receptor Signaling Pathway(TLR)	Hsa04620	13	0.013
Natural Killer Cell Mediated Cytotoxicity(NK)	Hsa04650	15	0.029

**For advanced lesions**			

Focal Adhesion(FA)	Hsa04510	25	0.000
ECM-Receptor Interaction(ECM)	Hsa04512	15	0.000
Toll-Like Receptor Signaling Pathway(TLR)	Hsa04620	13	0.002
Regulation of Actin Cytoskeleton(RAC)	Hsa04810	20	0.005
Fc Epsilon RI Signaling Pathway(FER)	Hsa04664	10	0.012
MAPK Signaling Pathway(MAPK)	Hsa04010	22	0.019
Natural Killer Cell Mediated Cytotoxicity(NK)	Hsa04650	13	0.021
Long-Term Potentiation(LTP)	Hsa04720	8	0.030
Cell Communication(CC)	Hsa01430	12	0.032
B Cell Receptor Signaling Pathway(BCR)	Hsa04662	8	0.042

Genes identified from SAM under FDR<1% were tested for overrepresentation within the pathways for the KEGG under the modified Fisher Exact test assumption; *p*-Value and genes involved in the pathway are provided for each pathway

### Protein validation of TLR7 expression

**Figure 9:**
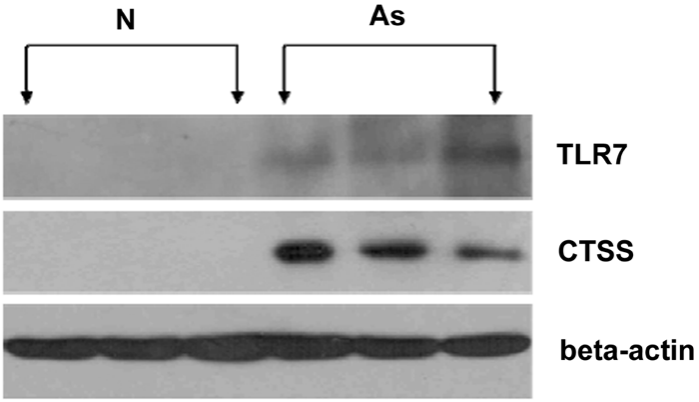
**Significant expression of TLR7 in femoral atherosclerotic lesions**. Western blot analysis of TLR7 in atherosclerotic femoral arteries (As, n = 3) and normal femoral arteries (N, n = 3). The protein level of Cathepsin S, whose expression was previously validated in atherosclerosis, was also examined in femoral atherosclerotic lesions. Beta – actin served as a loading control.

Members of the Toll receptor family are key mediators of innate immunity. They respond to various pathogen-associated stimuli and transduce complex signaling responses that are required for inflammation and for the subsequent development of adaptive immunity [26]. In atherosclerosis, TLR-mediated signaling cascades are observed in macrophages, mast cells and endothelial cells [27,28]. Data shown in this setting demonstrate that genes involved in TLR-mediated pathway are significantly up-regulated in intermediate or advanced lesions, including *TLR1, TLR2, TLR7*, and *MyD88*. *TLR1 *and *TLR2 *have been previously reported to be significantly regulated in atherosclerosis and their functional roles have been widely investigated in atherosclerosis [29,30]. However, the expression of *TLR7 *in atherosclerosis has not been reported before. TLR7 mediates innate responses by recognizing oligonucleotide based (RNA-) molecular patterns in endocytic compartments. Our data show that it is significantly up-regulated in both intermediate and advanced lesions. Western-blot analysis was performed to further validate its expression on the protein level (Figure 9). The function of TLR7 in atherogenesis is currently under further investigation.

## Discussion

In the present study, we first examined the gene expression profiles of PAD. Data analysis identified a number of genes that might be significantly correlated with different levels of PAD severity. The list of differentially expressed genes in intermediate and advanced lesions contains many genes which can be important for atherosclerosis. Most of these genes have not been reported to be related to atherosclerosis before. For example, *MAP4K4 *is a member of the serine/threonine protein kinase family. It has been shown to specifically activate MAPK8/JNK and mediate the TNF-alpha signaling pathway [31,32]. In this study, it was significantly and consistently up-regulated in both intermediate and advanced lesions.

A large multidisciplinary study is currently underway to comprehensively assess PAD at multiple levels [33], The goal of that study is to investigate 300 symptomatic patients with PAD undergoing medical management with or without vascular intervention by lower extremity angioplasty/stenting or vein graft bypass, and to test the hypothesis that the systemic inflammatory response after vascular intervention influences the local milieu responsible for vascular repair and adaptation [33]. Identification of genes through the work may be significant in the selection of candidate genes that can be investigated through these cases-control genetic epidemiology studies. Our research supports the idea that immune responses play a key role in the development of PAD.

In this report, immune related genes were shown to be significantly expressed during the development of PAD. Gene functional analysis further revealed that immune related categories and pathways were significant enriched in the different stages of PAD. In these immune related genes, several genes have been shown to modulate the development of atherosclerosis in mice models. For example, IgG Fc receptors (FcgammaRs) play a role in activating the immune system and in maintaining peripheral tolerance. Previous research suggested that *Fc*γ receptor deficiency protects against atherosclerosis in Apolipoprotein-E knockout mice [34]. The results suggest that broad-range inhibitors of immune and inflammatory responses can be considered as potential targets for the treatment of PAD. However, gene expression patterns of immune related genes can be different in different stages of PAD. For example, in intermediate lesions, MHC class II molecules were significantly up-regulated including *HLA-DMA, HLA-DMB, HLA-DPB1, HLA-DQB1, HLA-DRA, HLA-DRB1 and HLA-DRB5*. MHC class II molecules are normally restricted to a subset of antigen presenting dendritic cells, B cells, macrophages, and thymic epithelium cells [35]. These cells can be detected close to CD4+ T cells and present peptides to the T cells. The results suggest that there can be an ongoing immune activation in the intermediate lesions. However, MHC class II molecules were not differentially expressed in advanced lesions, even with a higher false discovery rate, which may suggest that the HLA-mediated immune activation may occur mainly in the progression stages of PAD. In addition, complement molecules were also significantly up-regulated in intermediate lesions, not in advanced lesions. Previous studies have implicated that activation of the complement system is probably associated with the initiation and progression of atherosclerosis [36,37]. Our data thus provide direct evidence from clinical samples demonstrating that complement system mainly play a role in the development stages of PAD. It is therefore conceivable that different and complex immune/inflammatory responses may take place at different stages of PAD.

Atherosclerosis is a systemic, multifocal disease leading to various symptoms and clinical events including cardiovascular disease, cerebrovascular disease, and peripheral arterial disease. Our results reveal that many genes identified in the report are also expressed in coronary or carotid atherosclerotic lesions. For example, *C3AR1 *and *C5R1 *are receptors of C3 (C3a) and C5a respectively. A recent study shows that *C3AR1 *and *C5R1 *are expressed in human atherosclerotic coronary plaques [38]. Double immunofluorescence staining has shown that the plaque of cells that express both *C3aR *and *C5aR *are macrophages, T cells, endothelial cells, and sub-endothelial smooth muscle cells. In addition, gene expression changes between atherosclerosis from coronary and carotid artery samples have been measured by microarray technology in recent years. One study using microarray found that 82 genes were differentially expressed in both animal model and human coronary artery atherosclerosis disease [39]. Our data confirmed 29 genes and 18 genes had significantly different expression in intermediate lesions and advanced lesions, respectively. Moreover, these genes had expression trends similar to the ones found in our data, but our data showed higher fold-changes. In these overlapping genes, 14 were reported to be involved in immune response. Another microarray study found that 206 genes were differentially expressed in aortic atherosclerosis samples [40]. Our data confirms 43 genes and 32 genes had significantly different expression in intermediate and advanced lesions (FDR<1%), respectively. Importantly, in these overlapping genes, 15 were reported to be involved in immune response. Taken together, the results suggested that immune response is a common feature in atherosclerosis-related diseases. Our microarray study differs from prior microarray studies in the array type, sample type, sample classification, and analytical techniques. Nevertheless, the high level of overlapping genes suggests that there are similar molecular mechanisms in the development of peripheral arterial disease and other atherosclerosis-related diseases.

Several limitations of our approach should be noted. First, hybridization-based microarrays, despite their immense potential, have inherent shortcomings related to deficient standardization of methods employed in normalization, statistical analysis, and so on [41,42]. In this study, we have attempted to limit these shortcomings by selecting subjects who were phenotypically similar to each other except for hypertension. In addition, the initial phases of data analysis, we used different normalization and statistical methods to identify differentially expressed genes. After choosing SAM, we used a rigorous false discovery rate to minimize false positive results. Expression patterns were validated by confirming mRNA expression patterns with conventional molecular techniques. We attempted, based on current literature, to suggest a potential functional role for genes whose expression was markedly altered. Second, atherosclerosis is a slow, progressive disease that may start in childhood; entirely normal arteries can only be obtained from young donors, a factor that can affect gene expression measurements. Although previous research and our data analysis suggest that age had very little effect on genes, further work is needed to identify age-related genes. Third, the relatively small number of patients did not allow us to assess serial changes in the disease development in more detail as would have been possible in animal models [36]. Furthermore, we do not know to what extent the observed changes in gene expression translate into protein synthesis and function, and which genes cause atherosclerosis. Future studies are needed to address these issues.

## Conclusion

We first examined the gene expression profiles of PAD; the results from this analysis provide an initial step towards a better understanding of molecular mechanisms underlying PAD development. Differences in immune-related responses were observable at the gene expression level. These findings may be significant for understanding the molecular basis of PAD and investigating pharmacological approaches for the prevention and amelioration of atherosclerosis in PAD.

## Methods

### Tissue Harvest

After obtaining informed consent, primary femoral artery specimens containing atherosclerotic lesions were taken from 30 patients undergoing surgical bypass or limb amputation at Shanghai Ninth People's Hospital. The specimens were immediately rinsed once with PBS and cut longitudinally by the surgeon. Three quarters of the samples were stored at once in – 80°C for subsequent total RNA extraction. The remaining samples were embedded in OCT medium and snap frozen for further morphological analysis. Clinical patient parameters were also registered. For controls, five normal femoral arteries were obtained from healthy donors during organ transplantation (male, mean 31.6 years; range 22–45 years). These five samples were without clinical or gross macroscopic signs of atherosclerotic disease. The Local Ethical Committee approved all procedures in this investigation, and proper protocol was followed throughout the entire course of the experiment.

### Histology

For each sample, cryostat sections of 8 um were stained with hematoxylin for 10 min and eosin for 2 min, dehydrated in graded alcohol, and cover-slipped with permanent mounting solution after xylene clearing.

### RNA Isolation and Quantification

Total RNA was isolated from the samples using a Trizol reagent (Invitrogen, Carlsbad, CA) and cleaned up using RNeasy Micro Kit (Qiagen, Valencia, CA) techniques. In brief, for each tissue, at least 100 mg sample was pulverized under liquid nitrogen. After complete disruption of the tissues, the Trizol reagent was added in the amount of 1 ml/100 mg. Total RNA was extracted using the protocol supplied with the Trizol reagent. After isolation, the RNA was cleaned up using the RNeasy Micro Kit. To remove any contaminating genomic DNA, a DNase step was included, following the manufacturer's protocol. The RNA quantity and quality were determined by an Agilent Bioanalyzer 2100 and an Eppendorf Biophotometer. Any RNA samples that showed degradation was excluded from the study.

### Microarray Experiment

One microgram of total RNA was used for generating biotin labeled cRNA. The labeling reaction was performed according to the standard Affymetrix^® ^protocol to generate a biotin-labeled cRNA probe. The samples were hybridized to the Affymetrix^® ^Human Genome -U133A Genechip, stained, washed and scanned according to the standard Affymetrix^® ^protocol. The computer data files to be used in data analysis (*.dat, *.cel, *.chp) were generated with the Affymetrix GeneChip Operating Software (GCOS) Version 1.4 (Affymetrix^®^), using the statistical algorithm provided. All chip samples were scanned using the same instrument and followed the same protocol. Data quality assessment was then performed following the guidance in Affymetrix data analysis fundamentals manual. All quality control results met Affymetrix recommended criteria.

### Data process and analysis

The probe level intensity data were transferred to ArrayAssist^® ^Software (StrataGene; La Jolla, CA) for further analysis. For comparison of differential gene expression between different stage groups, the background was removed and data were normalized in accordance to the GC-RMA method [43]. GC-RMA takes into account the GC content of the probe sequences when comparing the expression intensities of the different probesets. Then, the processed gene expression data were transformed into log base 2 and filtered to delete the genes whose detection calls were "absent" in all samples.

Microarray data analysis was carried out to identify individual genes that were significantly expressed between classes by the software package SAM (please see Availability & requirements for more information), using Δ = 0.5. Results from the difference analysis were clustered and displayed using the Cluster3.0 and Treeview1.1.0 software (please see Availability & requirements for more information). Each list of differentially expressed genes was analyzed in the context of Gene Ontology (GO) in order to identify groups of genes with similar functions, or processed using MAPPFinder (Gene MicroArray Pathway Profiler; please see Availability & requirements for more information). For each gene ontology term, the probability values were computed based on a hypergeometric distribution test by comparing (a) the number of genes annotated by the gene ontology term in a given list of differentially expressed genes with (b) the expected number of such genes. *Z-score>0 and p*-values < 0.05 were considered significant categories.

Similar methods were used to identify curated pathways that were significantly over-represented in the data using KEGG database by using DAVID (please see Availability & requirements for more information). For each pathway, the probability values were computed based on a modified Fisher exact test. EASE *p*-values < 0.05 were considered significant categories. The enriched pathways are not entirely separate from one another. For example, many genes involved in MAPK signaling pathway can also be involved in other pathways, such as NK pathway. The interconnectedness information was manually extracted from the pathway. Because the nature and complexity of these interactions varied from pathway to pathway, a simple line connecting two pathways was used to represent their interaction. The interaction map was generated for the interaction of enriched pathways using CytoScape software.

Transcription factor enrichment analysis was also performed. The putative targets of transcription factors from TRANSFAC (v7.4) were discovered by Xie et al [44] and downloaded from the supplementary web site (please see Availability & requirements for more information). All the RefSeq IDs were converted to Entrez Gene ID according to the mapping table downloaded from NCBI web site (please see Availability & requirements for more information). Enrichment of transcription factor targets was performed as described previously [45]. The interaction map was generated for the interaction of enriched transcription factors and their putative target genes using CytoScape software

### Real-time QPCR Analysis

One microgram of total RNA was reverse transcripted using random hexamers and superscript -II reverse transcriptase (Invitrogen, Carlsbad, CA). QPCR was performed by using ABI prism 7900 (ABI, Foster City, CA) and SYBR Green Detection (Toyobo, Japan). Primers were designed by using the Primer Express 2.0 software and verified by using a BLAST search. Sequences of the primers are listed [see Additional file 12]. The experimental conditions followed the manufacturer's protocol and the data were analyzed with sequence Detection Software 2.0 (ABI, Foster City, CA). Relative expression of mRNA was calculated with the comparative CT method. To standardize the amount of input RNA, the GAPDH gene was included. For each sample, the experiment was performed in triplicate.

### Western Blotting

Proteins were extracted after RNA isolation according to the Introvigen protocol (Invitrogen, Carlsbad, CA) and measured using a Bio-Rad DC protein assay (Bio-Rad, Richmond, CA, USA). Aliquots of protein (100 μg of protein each) were resolved on a 10% SDS-PAGE gel and transferred to a polyvinylidene difluoride membrane (Millipore, Medford, MA, USA). The membrane was incubated with a primary antibody overnight at 4°C and then with a secondary antibody conjugated with alkaline phosphatase (1 h at room temperature), which was detected by a chemiluminescence method. The following polyclonal primary antibodies were used: anti-human TLR7 (1:300, IMGENEX, San Diego, CA), anti-human CTSS (1:400, Abcam Inc), anti-human beta-action (1:10000, Abcam Inc).

### Statistics

The statistical significance of real-time results was examined with the nonparametric Mann-Whitney test, using GraphPad Prism 4. In the experiment, *p *values < 0.05 were considered significantly different between the lesions group and the normal artery group.

## Availability & requirements

SAM software package: 

Cluster3.0 and Treeview1.1.0 software: 

MAPPFinder (Gene MicroArray Pathway Profiler): 

DAVID: 

Xie et al Supplementary Information: 

NCBI web site: 

## Authors' contributions

SJF, experiment design and conduction, and manuscript drafting; HGZ, clinical sample processing; JTS, transcription factor enrichment analysis; AA, WPK, KC and LO–M, data analysis and manuscript revising; JQZ, data analysis; YZD, data analysis and literature support; JZ and MEJ, experimental design and manuscript revising; JGJ, experiment design, data analysis and manuscript revising. All the authors read and approved this version of the manuscript.

## Supplementary Material

Additional File 1 **Table 1**. 366 differentially expressed genes in intermediate lesions relative to normal femoral arteries. Affymetrix Probe Set ID, Gene Title, Gene Symbol, GO Biological Process, GO Molecular Function, GO Cellular Component, Unigene, Entrez Gene, Ensembl, Chromosome Number, Socre(d), Fold Change and q-value(%) which is the lowest FDR are listed in the table. Click here for file

Additional File 2 **Table 2**. Immune-related genes in intermediate lesions relative to normal femoral arteries, Affymetrix Probe Set ID, Gene Title, Gene Symbol, GO Biological Process, GO Molecular Function, GO Cellular Component, Unigene, Entrez Gene, Ensembl, Chromosome Number, Socre(d), Fold Change and q-value(%) which is the lowest FDR are listed in the table. Click here for file

Additional File 3 **Table 3**. Cell component and molecular function categories overrepresented in intermediate lesions, the first half of the table indicates categories highly significant for up-regulated genes; the second half of the table shows categories highly significant for down-regulated genes. The calculated *p*-values and Z-scores for each category are shown. Click here for file

Additional File 4 **Table 4**. 447 differentially expressed genes in advanced lesions relative to normal femoral arteries. Affymetrix Probe Set ID, Gene Title, Gene Symbol, GO Biological Process, GO Molecular Function, GO Cellular Component, Unigene, Entrez Gene, Ensembl, Chromosome Number, Socre(d), Fold Change and q-value(%) which is the lowest FDR are listed in the table. Click here for file

Additional File 5 **Table 5**. Immune-related genes in advanced lesions relative to normal femoral arteries, Affymetrix Probe Set ID, Gene Title, Gene Symbol, GO Biological Process, GO Molecular Function, GO Cellular Component, Unigene, Entrez Gene, Ensembl, Chromosome Number, Socre(d), Fold Change and q-value(%) which is the lowest FDR are listed in the table. Click here for file

Additional File 6 **Table 6**. Cell component and molecular function categories overrepresented in advanced lesions, the first half of the table indicates categories highly significant for up-regulated genes; the second half of the table shows categories highly significant for down-regulated genes. The calculated *p*-values and Z-scores for each category are shown. Click here for file

Additional File 7 **Table 7**. 68 commonly up-regulated genes in intermediate lesions and advanced lesions, Affymetrix Probe Set ID, Gene Title, Gene Symbol, GO Biological Process, GO Molecular Function, GO Cellular Component, Unigene, Entrez Gene, Ensembl, Chromosome Number, Socre(d), Fold Change and q-value(%) which is the lowest FDR are listed in the table. Click here for file

Additional File 8 **Table 8**. 614 differentially expressed genes in disease progression. Affymetrix Probe Set ID, Cluster ID, Gene Title, Gene Symbol, GO Biological Process, GO Molecular Function, GO Cellular Component, Unigene, Entrez Gene, Ensembl, Chromosome Number, Socre(d), Contrast which is the standardized mean difference between the gene's expression in that class versus its overall mean expression, and q-value(%)which is the lowest FDR are listed. In the table, contrast1, 2 and 3 represent the standardized mean difference in normal femoral arteries, intermediate lesions, and advanced lesions, respectively. Click here for file

Additional File 9 **Table 9**. The detail GO overrepresented categories for disease progression in each cluster, the calculated *p*-values and Z-scores for each category are shown in the table. Click here for file

Additional File 10 **Table 10**. 72 putative AP-1 target genes in disease progression. Affymetrix Probe Set ID, Gene Title, Gene Symbol, GO Biological Process, GO Molecular Function, GO Cellular Component, Unigene, Entrez Gene, Ensembl, Chromosome Number, Socre(d), Contrast, and q-value(%) are listed. Click here for file

Additional File 11 **Table 11**. 55 putative CREB target genes in disease progression. Affymetrix Probe Set ID, Gene Title, Gene Symbol, GO Biological Process, GO Molecular Function, GO Cellular Component, Unigene, Entrez Gene, Ensembl, Chromosome Number, Socre(d), Contrast, and q-value(%) were listed. Click here for file

Additional File 12 **Table 12**. The primer sequences of selected genes for real-time PCR. Click here for file
