# Supplementary material for: Peripheral arterial occlusive disease: Global gene expression analyses suggest a major role for immune and inflammatory responses
Source: BMC Genomics. 2008 Aug 1;9:369. doi: 10.1186/1471-2164-9-369 (PMC2529314; doi:10.1186/1471-2164-9-369)
Supplement: Additional File 3 — Table 3. Cell component and molecular function categories overrepresented in intermediate lesions, the first half of the table indicates categories highly significant for up-regulated genes; the second half of the table shows categories highly significant for down-regulated genes. The calculated p-values and Z-scores for each category are shown. [file 1471-2164-9-369-S3.doc]

**Table3**- Cell component and molecular function categories overrepresented in intermediate lesions

| **GO type** | **GO Name** | **Z-score** | ***P*-value** |
| --- | --- | --- | --- |
| **For up-regulated genes** | | | |
| Cell component | hemoglobin complex | 5.102 | 0.009 |
| Cell component | Golgi membrane | 2.678 | 0.031 |
| Cell component | vacuole | 6.593 | 0.000 |
| Cell component | lysosome | 6.027 | 0.000 |
| Cell component | membrane | 2.9 | 0.004 |
| Cell component | integral to membrane | 3.787 | 0.001 |
| Cell component | integral to plasma membrane | 2.057 | 0.042 |
| Cell component | plasma membrane | 3.289 | 0.000 |
| Molecular function | antigen binding | 3.524 | 0.014 |
| Molecular function | carbohydrate binding | 2.841 | 0.018 |
| Molecular function | heparin binding | 4.039 | 0.005 |
| Molecular function | peptidoglycan binding | 7.477 | 0.002 |
| Molecular function | anaphylatoxin receptor activity | 8.709 | 0.002 |
| Molecular function | IgG binding | 6.629 | 0.003 |
| Molecular function | chemokine activity | 2.961 | 0.016 |
| Molecular function | G-protein-coupled receptor binding | 2.633 | 0.033 |
| Molecular function | integrin binding | 3.608 | 0.018 |
| Molecular function | deaminase activity | 3.214 | 0.033 |
| Molecular function | MAP kinase phosphatase activity | 4.767 | 0.009 |
| Molecular function | carboxypeptidase activity | 3.524 | 0.021 |
| Molecular function | dipeptidyl-peptidase activity | 4.767 | 0.006 |
| Molecular function | signal transducer activity | 5.923 | 0.000 |
| Molecular function | pattern recognition receptor activity | 7.839 | 0.001 |
| Molecular function | transmembrane receptor activity | 2.981 | 0.004 |
| Molecular function | MHC class II receptor activity | 6.25 | 0.001 |
| Molecular function | receptor signaling protein activity | 3.929 | 0.002 |
| Molecular function | oxygen transporter activity | 4.015 | 0.016 |
|  | | | |
| **For down-regulated genes** | | | |
| Cell component | cytoplasm | 2.028 | 0.030 |
| Cell component | cytosol | 2.267 | 0.042 |
| Cell component | glycine cleavage complex | 11.038 | 0.000 |
| Cell component | mitochondrion | 2.277 | 0.034 |
| Cell component | small nucleolar ribonucleoprotein complex | 4.286 | 0.015 |
| Cell component | DNA-directed RNA polymerase II\, holoenzyme | 3.254 | 0.022 |
| Cell component | transcription factor complex | 3.494 | 0.017 |
| Cell component | organelle lumen | 3.558 | 0.003 |
| Molecular function | translation factor activity\, nucleic acid binding | 2.336 | 0.045 |
| Molecular function | transforming growth factor beta receptor binding | 7.678 | 0.000 |
| Molecular function | unfolded protein binding | 3.114 | 0.014 |
| Molecular function | aminomethyltransferase activity | 8.457 | 0.003 |
| Molecular function | nucleotide kinase activity | 4.444 | 0.013 |
| Molecular function | DNA-directed RNA polymerase activity | 2.85 | 0.045 |
| Molecular function | translation regulator activity | 2.285 | 0.047 |
| Molecular function | hydrogen ion transporter activity | 2.336 | 0.036 |
