# Supplementary material for: Peripheral arterial occlusive disease: Global gene expression analyses suggest a major role for immune and inflammatory responses
Source: BMC Genomics. 2008 Aug 1;9:369. doi: 10.1186/1471-2164-9-369 (PMC2529314; doi:10.1186/1471-2164-9-369)
Supplement: Additional File 6 — Table 6. Cell component and molecular function categories overrepresented in advanced lesions, the first half of the table indicates categories highly significant for up-regulated genes; the second half of the table shows categories highly significant for down-regulated genes. The calculated p-values and Z-scores for each category are shown. [file 1471-2164-9-369-S6.doc]

**Table 6**- Cell component and molecular function categories overrepresented in advanced lesions

| **GO Type** | **GO Name** | **Z-score** | ***P*-value** |
| --- | --- | --- | --- |
| **For up-regulated genes** | | | |
| Cell component | integral to plasma membrane | 2.537 | 0.012 |
| Cell component | plasma membrane | 2.564 | 0.008 |
| Cell component | vacuole | 3.445 | 0.011 |
| Cell component | lysosome | 3.596 | 0.007 |
| Cell component | hemoglobin complex | 6.011 | 0.000 |
| Cell component | extracellular matrix | 3.959 | 0.000 |
| Cell component | extracellular matrix (sensu Metazoa) | 4.011 | 0.000 |
| Cell component | collagen | 7.711 | 0.000 |
| Cell component | collagen type VI | 12.498 | 0.000 |
| Cell component | collagen type I | 10.14 | 0.000 |
| Cell component | extracellular region | 2.688 | 0.010 |
| Molecular function | oxygen binding | 2.788 | 0.048 |
| Molecular function | integrin binding | 2.718 | 0.046 |
| Molecular function | heparin binding | 2.634 | 0.043 |
| Molecular function | extracellular matrix structural constituent | 3.381 | 0.011 |
| Molecular function | structural constituent of bone | 8.725 | 0.000 |
| Molecular function | acidic amino acid transporter activity | 6.468 | 0.003 |
| Molecular function | L-amino acid transporter activity | 3.616 | 0.034 |
| Molecular function | lipid transporter activity | 4.971 | 0.002 |
| Molecular function | dicarboxylic acid transporter activity | 5.629 | 0.005 |
| Molecular function | oxygen transporter activity | 4.778 | 0.003 |
| Molecular function | anion\:cation symporter activity | 3.018 | 0.045 |
| Molecular function | deaminase activity | 6.05 | 0.001 |
| Molecular function | caspase activity | 7.363 | 0.000 |
| Molecular function | enzyme regulator activity | 3.772 | 0.003 |
| Molecular function | protease inhibitor activity | 3.445 | 0.011 |
| Molecular function | cysteine protease inhibitor activity | 3.616 | 0.020 |
| Molecular function | GTPase regulator activity | 2.711 | 0.024 |
| Molecular function | guanyl-nucleotide exchange factor activity | 2.851 | 0.023 |
| Molecular function | small GTPase regulator activity | 3.098 | 0.017 |
| Molecular function | signal transducer activity | 4.013 | 0.000 |
| Molecular function | scavenger receptor activity | 3.616 | 0.016 |
|  | | | |
| **For down-regulated genes** | | | |
| Cell component | integrin complex | 3.233 | 0.019 |
| Cell component | stress fiber | 9.762 | 0.000 |
| Molecular function | magnesium ion binding | 3.775 | 0.001 |
| Molecular function | RNA binding | 2.535 | 0.021 |
| Molecular function | heat shock protein binding | 6.188 | 0.000 |
| Molecular function | transforming growth factor beta receptor binding | 5.401 | 0.004 |
| Molecular function | unfolded protein binding | 5.212 | 0.000 |
| Molecular function | vitamin transporter activity | 5.98 | 0.003 |
| Molecular function | cofactor transporter activity | 4.265 | 0.022 |
| Molecular function | prenyltransferase activity | 3.389 | 0.026 |
| Molecular function | calmodulin regulated protein kinase activity | 2.946 | 0.034 |
| Molecular function | phosphoric monoester hydrolase activity | 2.771 | 0.019 |
| Molecular function | protein serine/threonine phosphatase activity | 3.15 | 0.021 |
| Molecular function | ligase activity\, forming carbon-sulfur bonds | 3.389 | 0.045 |
| Molecular function | fatty-acid ligase activity | 4.575 | 0.015 |
| Molecular function | long-chain-fatty-acid-CoA ligase activity | 4.575 | 0.015 |
| Molecular function | serine-type endopeptidase inhibitor activity | 2.524 | 0.027 |
