# Supplementary material for: Peripheral arterial occlusive disease: Global gene expression analyses suggest a major role for immune and inflammatory responses
Source: BMC Genomics. 2008 Aug 1;9:369. doi: 10.1186/1471-2164-9-369 (PMC2529314; doi:10.1186/1471-2164-9-369)
Supplement: Additional File 9 — Table 9. The detail GO overrepresented categories for disease progression in each cluster, the calculated p-values and Z-scores for each category are shown in the table. [file 1471-2164-9-369-S9.doc]

**Table 9- The detail GO overrepresented categories for disease progression in each cluster**

| **GO Type** | **GO Name** | **Z-Score** | ***P*-value** |
| --- | --- | --- | --- |
| **Cluster I** | | | |
| Biological process | immune response | 8.908 | 0.000 |
| Biological process | inflammatory response | 7.643 | 0.000 |
| Biological process | myeloid leukocyte mediated immunity | 5.889 | 0.004 |
| Biological process | mast cell mediated immunity | 6.657 | 0.003 |
| Biological process | defense response | 6.830 | 0.000 |
| Biological process | cellular defense response | 5.938 | 0.001 |
| Biological process | lymphocyte proliferation | 6.673 | 0.000 |
| Biological process | mast cell degranulation | 6.657 | 0.003 |
| Biological process | response to stimulus | 5.345 | 0.000 |
| Biological process | chemotaxis | 7.611 | 0.000 |
| Biological process | leukocyte migration | 3.254 | 0.025 |
| Biological process | neutrophil chemotaxis | 5.257 | 0.005 |
| Biological process | response to biotic stimulus | 2.940 | 0.014 |
| Biological process | response to chemical stimulus | 3.714 | 0.001 |
| Biological process | response to external stimulus | 5.981 | 0.000 |
| Biological process | response to stress | 3.414 | 0.002 |
| Biological process | leukocyte differentiation | 2.457 | 0.035 |
| Biological process | myeloid cell differentiation | 4.458 | 0.002 |
| Biological process | cell surface receptor linked signal transduction | 2.937 | 0.009 |
| Biological process | cytokine and chemokine mediated signaling pathway | 4.378 | 0.004 |
| Biological process | enzyme linked receptor protein signaling pathway | 2.594 | 0.023 |
| Biological process | epidermal growth factor receptor signaling pathway | 2.655 | 0.048 |
| Biological process | intracellular signaling cascade | 4.288 | 0.000 |
| Biological process | protein kinase cascade | 4.716 | 0.000 |
| Biological process | JAK-STAT cascade | 3.254 | 0.020 |
| Biological process | tyrosine phosphorylation of STAT protein | 4.194 | 0.017 |
| Biological process | MAPKKK cascade | 3.062 | 0.006 |
| Biological process | second-messenger-mediated signaling | 2.681 | 0.024 |
| Biological process | cytokine secretion | 5.257 | 0.000 |
| Biological process | endocytosis | 1.956 | 0.049 |
| Biological process | alkene metabolic process | 4.500 | 0.012 |
| Biological process | icosanoid biosynthetic process | 2.768 | 0.046 |
| Biological process | peptidyl-tyrosine phosphorylation | 4.003 | 0.006 |
| Biological process | angiogenesis | 4.452 | 0.000 |
| Biological process | hemopoietic or lymphoid organ development | 4.378 | 0.000 |
| Biological process | multicellular organismal development | 2.204 | 0.028 |
| Biological process | oxygen transport | 5.316 | 0.005 |
| Biological process | phosphate transport | 2.951 | 0.019 |
| Biological process | sequestering of metal ion | 4.500 | 0.006 |
| Biological process | cell adhesion | 4.580 | 0.000 |
| Biological process | cell homeostasis | 3.450 | 0.003 |
| Cell component | hemoglobin complex | 5.889 | 0.007 |
| Cell component | endoplasmic reticulum lumen | 2.768 | 0.049 |
| Cell component | lysosome | 3.436 | 0.004 |
| Cell component | integral to plasma membrane | 3.908 | 0.001 |
| Cell component | immunological synapse | 3.506 | 0.028 |
| Cell component | plasma membrane | 4.899 | 0.000 |
| Cell component | collagen | 3.085 | 0.025 |
| Cell component | extracellular region | 2.888 | 0.007 |
| Cell component | receptor complex | 4.804 | 0.000 |
| Molecular function | anion binding | 2.932 | 0.030 |
| Molecular function | peptide binding | 3.828 | 0.004 |
| Molecular function | cytokine binding | 3.715 | 0.007 |
| Molecular function | molecular adaptor activity | 3.284 | 0.008 |
| Molecular function | insulin receptor binding | 3.506 | 0.046 |
| Molecular function | G-protein-coupled receptor binding | 4.657 | 0.000 |
| Molecular function | chemokine receptor binding | 5.549 | 0.000 |
| Molecular function | immunoglobulin receptor activity | 7.771 | 0.002 |
| Molecular function | rhodopsin-like receptor activity | 2.351 | 0.034 |
| Molecular function | scavenger receptor activity | 4.629 | 0.002 |
| Molecular function | deaminase activity | 2.890 | 0.036 |
| Molecular function | caspase activity | 7.185 | 0.000 |
| Molecular function | endopeptidase activity | 2.341 | 0.020 |
| Molecular function | exopeptidase activity | 2.459 | 0.043 |
| Molecular function | protein disulfide isomerase activity | 4.866 | 0.012 |
| Molecular function | receptor signaling protein tyrosine kinase activity | 3.933 | 0.020 |
| Molecular function | extracellular matrix structural constituent | 2.757 | 0.029 |
| Molecular function | amino acid-polyamine transporter activity | 4.194 | 0.025 |
| Molecular function | oxygen transporter activity | 5.316 | 0.005 |
|  | | | |
| **Cluster Ⅱ** | | | |
| Biological process | apoptosis | 3.871 | 0.002 |
| Biological process | anti-apoptosis | 3.580 | 0.003 |
| Biological process | apoptotic nuclear changes | 3.212 | 0.026 |
| Biological process | DNA fragmentation during apoptosis | 3.712 | 0.021 |
| Biological process | cell structure disassembly during apoptosis | 3.075 | 0.032 |
| Biological process | cell cycle | 4.108 | 0.000 |
| Biological process | cell cycle arrest | 3.570 | 0.006 |
| Biological process | cell growth | 2.285 | 0.037 |
| Biological process | response to nutrient | 3.919 | 0.033 |
| Biological process | cell fate determination | 6.478 | 0.000 |
| Biological process | intracellular protein transport across a membrane | 2.726 | 0.045 |
| Biological process | protein import into nucleus\, translocation | 2.726 | 0.045 |
| Biological process | epidermal cell differentiation | 3.075 | 0.044 |
| Biological process | protein folding | 3.677 | 0.001 |
| Biological process | posttranslational protein folding | 5.545 | 0.004 |
| Biological process | RNA metabolic process | 5.378 | 0.000 |
| Biological process | transcription | 4.593 | 0.000 |
| Biological process | developmental process | 4.798 | 0.000 |
| Biological process | tissue morphogenesis | 3.522 | 0.012 |
| Biological process | organ development | 3.799 | 0.000 |
| Biological process | peripheral nervous system development | 2.950 | 0.046 |
| Biological process | multicellular organismal development | 3.927 | 0.000 |
| Biological process | vitamin transport | 4.426 | 0.008 |
| Biological process | macromolecule metabolic process | 3.022 | 0.005 |
| Biological process | primary metabolic process | 2.603 | 0.011 |
| Biological process | response to stimulus | 4.579 | 0.000 |
| Biological process | inflammatory response | 2.844 | 0.010 |
| Biological process | response to biotic stimulus | 4.466 | 0.000 |
| Biological process | response to chemical stimulus | 4.988 | 0.000 |
| Biological process | response to external stimulus | 4.198 | 0.000 |
| Biological process | response to wounding | 3.624 | 0.000 |
| Biological process | response to stress | 5.702 | 0.000 |
| Biological process | rhythmic process | 5.658 | 0.000 |
| Biological process | circadian rhythm | 5.090 | 0.001 |
| Cell component | intracellular membrane-bound organelle | 2.402 | 0.021 |
| Cell component | nucleus | 6.489 | 0.000 |
| Cell component | nuclear lumen | 2.339 | 0.021 |
| Cell component | membrane-bound organelle | 2.393 | 0.021 |
| Molecular function | carbohydrate binding | 2.306 | 0.040 |
| Molecular function | nucleic acid binding | 4.873 | 0.000 |
| Molecular function | transcription factor activity | 6.393 | 0.000 |
| Molecular function | heat shock protein binding | 3.718 | 0.008 |
| Molecular function | protein dimerization activity | 4.777 | 0.000 |
| Molecular function | NF-kappaB binding | 4.744 | 0.011 |
| Molecular function | transcription corepressor activity | 3.187 | 0.013 |
| Molecular function | unfolded protein binding | 4.824 | 0.000 |
| Molecular function | ATP-dependent RNA helicase activity | 4.894 | 0.005 |
| Molecular function | MAP kinase phosphatase activity | 4.744 | 0.005 |
| Molecular function | NAD+ ADP-ribosyltransferase activity | 3.212 | 0.027 |
| Molecular function | chaperone regulator activity | 5.124 | 0.011 |
| Molecular function | kinase inhibitor activity | 3.768 | 0.011 |
| Molecular function | steroid hormone receptor activity | 3.140 | 0.021 |
| Molecular function | cofactor transporter activity | 5.124 | 0.010 |
| Molecular function | vitamin transporter activity | 6.188 | 0.004 |
|  | | | |
| **Cluster Ⅲ** | | | |
| Biological process | neurotransmitter biosynthetic process | 4.153 | 0.010 |
| Biological process | neurotransmitter secretion | 2.483 | 0.048 |
| Biological process | synaptic transmission | 2.273 | 0.022 |
| Biological process | nerve growth factor receptor signaling pathway | 6.733 | 0.003 |
| Biological process | protein-RNA complex assembly | 2.256 | 0.044 |
| Biological process | axonogenesis | 3.005 | 0.011 |
| Biological process | neurogenesis | 2.612 | 0.014 |
| Biological process | cell motility | 2.093 | 0.042 |
| Biological process | neutrophil chemotaxis | 2.767 | 0.044 |
| Biological process | neuron recognition | 3.315 | 0.046 |
| Biological process | extracellular structure organization and biogenesis | 2.342 | 0.044 |
| Biological process | synapse organization and biogenesis | 2.559 | 0.037 |
| Biological process | glutamine family amino acid metabolic process | 2.559 | 0.049 |
| Biological process | glucan metabolic process | 2.999 | 0.025 |
| Biological process | glycogen metabolic process | 2.999 | 0.025 |
| Biological process | protein modification process | 3.166 | 0.001 |
| Biological process | pyrimidine nucleotide metabolic process | 2.901 | 0.035 |
| Biological process | RNA processing | 2.183 | 0.042 |
| Biological process | RNA splicing | 2.591 | 0.021 |
| Biological process | phosphate metabolic process | 2.346 | 0.024 |
| Biological process | dephosphorylation | 3.131 | 0.007 |
| Biological process | localization of cell | 2.093 | 0.042 |
| Biological process | biopolymer modification | 3.517 | 0.000 |
| Biological process | blood pressure regulation | 4.028 | 0.008 |
| Biological process | heart contraction | 2.901 | 0.022 |
| Cell component | filopodium | 3.550 | 0.019 |
| Cell component | cortical actin cytoskeleton | 2.767 | 0.041 |
| Cell component | actin cytoskeleton | 3.736 | 0.000 |
| Cell component | ubiquitin ligase complex | 2.397 | 0.046 |
| Cell component | protein serine/threonine phosphatase complex | 2.901 | 0.035 |
| Molecular function | magnesium ion binding | 2.588 | 0.027 |
| Molecular function | calmodulin binding | 3.740 | 0.001 |
| Molecular function | actinin binding | 5.747 | 0.001 |
| Molecular function | kinase binding | 2.500 | 0.041 |
| Molecular function | transforming growth factor beta receptor binding | 4.555 | 0.014 |
| Molecular function | vitamin binding | 2.267 | 0.041 |
| Molecular function | DNA-dependent ATPase activity | 2.410 | 0.048 |
| Molecular function | phosphoric ester hydrolase activity | 1.985 | 0.043 |
| Molecular function | ligase activity | 2.572 | 0.014 |
| Molecular function | small protein conjugating enzyme activity | 2.349 | 0.033 |
| Molecular function | calmodulin regulated protein kinase activity | 3.110 | 0.036 |
