# Supplementary material for: Peripheral arterial occlusive disease: Global gene expression analyses suggest a major role for immune and inflammatory responses
Source: BMC Genomics. 2008 Aug 1;9:369. doi: 10.1186/1471-2164-9-369 (PMC2529314; doi:10.1186/1471-2164-9-369)
Supplement: Additional File 12 — Table 12. The primer sequences of selected genes for real-time PCR. [file 1471-2164-9-369-S12.doc]

**Table 12-The primer sequences of selected genes for real-time PCR**

| Primer Name | Primer | Sequences(5'to3') |
| --- | --- | --- |
| ALOX5 | Forward Primer | CCCTTCGGATGCAAAATACG |
|  | Reverse Primer | CAGACACCAGATGTGTTCGCA |
| TLR7 | Forward Primer | GTGGAAATTGCCCTCGTTGT |
|  | Reverse Primer | TGTCAGCGCA TCAAAAGCAT |
| COL1A1 | Forward Primer | CCACCAATCACCTGCGTACA |
|  | Reverse Primer | CATCGCACAACACCTTGCC |
| CTSS | Forward Primer | GAACCTGGTGGATTGCTCAACT |
|  | Reverse Primer | TCGATGCCCTTGTTATCAATGA |
| SPP1 | Forward Primer | CAGACCCTTCCAAGTAAGTCCAAC |
|  | Reverse Primer | CGAGTCAATGGAGTCCTGGC |
| CXCR4 | Forward Primer | CTCCATCATCTTCTTAACTGGCATT |
|  | Reverse Primer | GTGCAGCCTGTACTTGTCCGT |
| GAPDH | Forward Primer | GAAGGTGAAGGTCGGAGTC |
|  | Reverse Primer | GAAGATGGTGATGGGATTTC |
